# Geologic factors leadingly drawing the macroecological pattern of rocky desertification in southwest China

**DOI:** 10.1038/s41598-020-58550-1

**Published:** 2020-01-29

**Authors:** Miao Jiang, Yi Lin, Ting On Chan, Yunjun Yao, Guang Zheng, Shezhou Luo, Lin Zhang, Daping Liu

**Affiliations:** 1 Institute of Mineral Resources Research, China Metallurgical Geology Bureau, Beijing, 101300 China; 2 School of Earth and Space Sciences, Peking University, Beijing, 100871 China; 3 School of Geography and Planning, Sun Yat-sen University, Guangzhou, 510275 China; 4 Faculty of Geographical Science, Beijing Normal University, Beijing, 100875 China; 5 International Institute for Earth System Science, Nanjing University, Nanjing, 210023 China; 6 College of Resources and Environment, Fujian Agriculture and Forestry University, Fuzhou, 350002 China; 7 School of Physics, Peking University, Beijing, 100871 China

**Keywords:** Geomorphology, Macroecology

## Abstract

Rocky desertification (RD) is a special process of land deterioration in karst topography, with a view of bedrock exposure and an effect of ecological degradation. Among the three largest karst regions in the world, southwest China boasts the largest RD area and highest diversity of karst landscapes. However, inefficient field surveying tends to restrict earlier studies of RD to local areas, and the high complexity of karst geomorphology in southwest China further lead to the shortage of the knowledge about its macroecological pattern so far. To address this gap, this study innovatively took county as the unit to statistically explore the links between the 2008-censused distributions of county-level RD in southwest China and its potential impact factors of three kinds (geologic, climatic, and anthropogenic), all transformed into the same mapping frame. Spatial pattern analyses based on spatial statistics and artificial interpretation unveiled the macroscopic characteristics of RD spatial patterns, and attribution analyses based on correlation analysis and dominance analysis exposed the links of the impact factors to RD and their contributions in deciding the macroscopic pattern of RD. The results suggested that geologic factors play a first role in drawing the macroecological pattern of RD, also for the slight-, moderate-, and severe-level RD scenarios, in southwest China. Despite this inference somehow collides with the popular awareness that anthropogenic factors like human activities are leadingly responsible for the RD-relevant losses, the findings are of practical implications in guiding making the macroscopic policies for mitigating RD degradation and advancing its environmental restoration.

## Introduction

Rocky desertification (RD) recently has attracted increasing academic and administrative attentions^1–3^, since this kind of unique land degradation phenomena has become one of the most serious ecological problems in the world. First proposed in the earlier 1980s^4^, the concept of RD is defined as a category of terrestrial processes of land deteriorations involving severe soil erosion, extensive exposure of bedrocks, drastic decrease of land productivity, and appearance of desert-like landscapes^5^. This adverse effect may trigger both natural hazards (such as droughts, floods, landslides, and land subsidence) and worsen economic, social, and cultural lives. At the regional scales, RD even can disturb climatic development and carbon balance^2^. During its studies, people found that the occurrences of RD are often related to karst geomorphology, which is a distinctive topography resulting from the solution process of acidic water acting on carbonate bedrocks^6^. Globally, karst landscapes cover ~22, 000, 000 km^2^, accounting for ~15% of the total terrestrial area^7^. Thereby, understanding of RD more comprehensively and mitigating its risks for such a large karst landform is of fundamental implications for advancing global ecological protection^8^.

**Figure 1:**
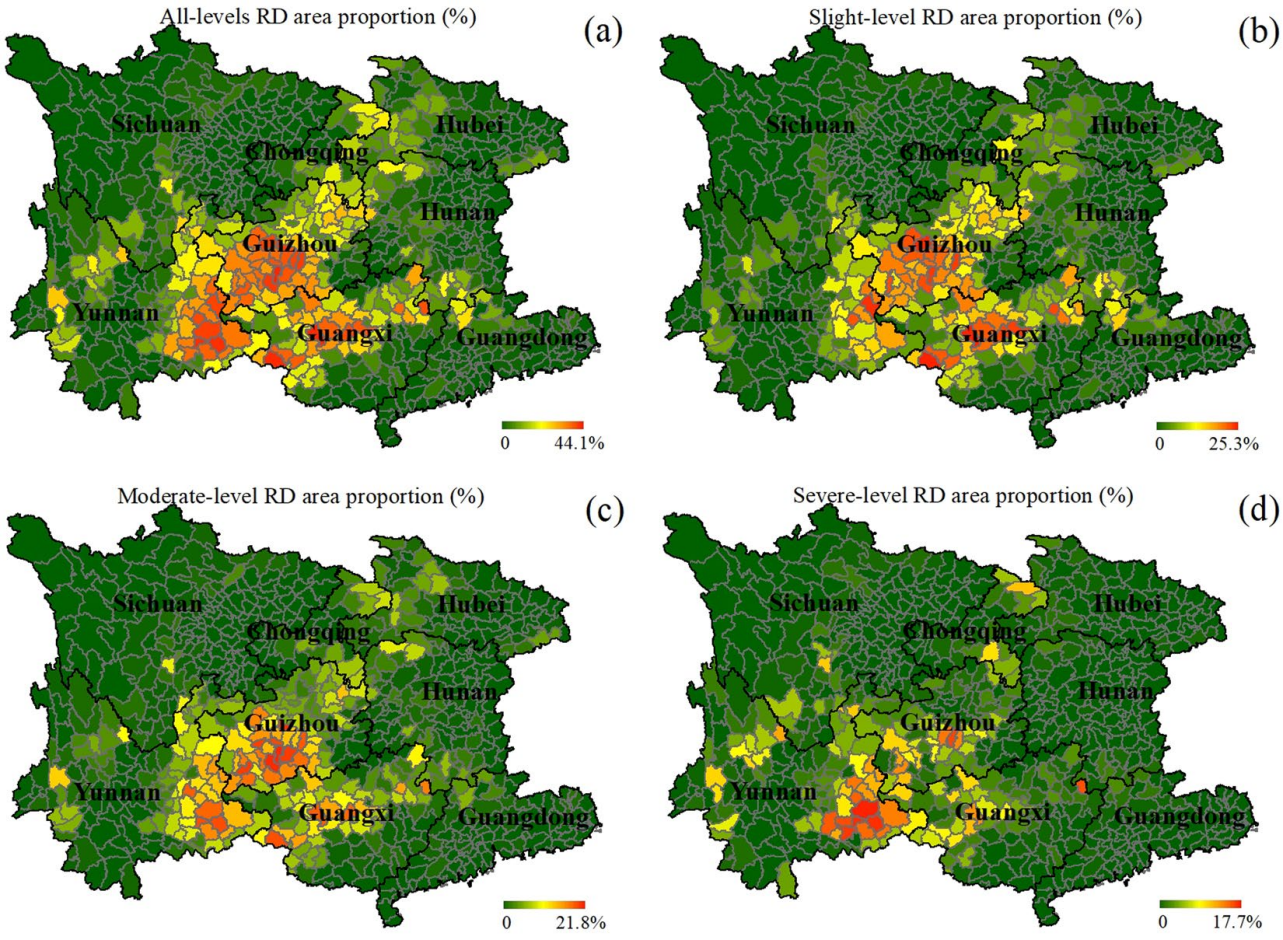
The study area of southwest China, comprising eight province-level municipalities – six provinces (Yunnan, Sichuan, Hubei, Hunan, Guangdong, and Guizhou), an autonomous region (Guangxi), and a city directly under the Central Government (Chongqing), with both their own and counties’ boundaries outlined and the county-based spatial patterns of the RD situations at the (**a**) all, (**b**) slight, (**c**) moderate and (**d**) severe levels marked.

Among the three largest karst regions in the world, southwest China comprising eight province-level municipalities (Fig. 1), including six provinces (Guangdong, Yunnan, Sichuan, Hubei, Hunan, and Guizhou), an autonomous region (Guangxi), and a city directly under the Central Government (Chongqing), is the most complex one, since it boasts the largest area of exposed carbonate and the most complete array of karst landscape categories^9^. This region boasts the most fragile ecological environment and the highest population density^10^. For the poverty in this region, RD is also considered as one of the reasons^11^. Consequently, for this region there has been a large amount of endeavors made by the central government, local governments, and research institutes to reduce its RD-relevant ecological deteriorations^12–14^. To date, experiences in recovering the typical RD areas suggested that it is essential to first identify the causes of RD, involving natural and anthropogenic factors, in order to probe the mechanism of RD formations at different spatial scales^11,15^. From the perspective of theoretical analysis, it is also preferred to first grasp the all-around RD causes for designing macroscopic policies to advance its preservation and restoration^16^.

However, earlier studies on RD causes in southwest China mostly focused on the natural or anthropogenic factors within local areas in a factor-individually or factors-limited way, as reflected by a short literature review^11,14,17–22^ as follows. Li *et al*.^11^ investigated the correlation between RD and lithology in the karst area of Guizhou. Wang *et al*.^17^ explored only the constraints of geological lithology on RD evolution in Guizhou as well. Xiong *et al*.^18^ studied the influence of both temperature and precipitation on RD in Yongshun County, Hunan. Yang *et al*.^19^ probed the effects of geology and landform on RD in Dahua County, Guangxi. Li *et al*.^20^ examined the relations between land use and RD in Panxian County, Guizhou. Zhang *et al*.^14^ attempted the approach of radial basis function network model to assess the RD situation in northwest Guangxi. Qi *et al*.^21^ found that soil pH is the primary factor correlating with soil microbiome in the karst RD areas in Wushan County, Chongqing. Bai *et al*.^22^ explored the dissolution mechanism and karst RD development of carbonate rocks in the karst area – Zhenfeng-Guanling-Huajiang County, Guizhou. Recently, increasing highlights have also been extended to the whole southwest China. Yan and Cai^23^ checked multi-scale anthropogenic driving forces involving RD in southwest China. Zhang *et al*.^24^ investigated the social and economic influencing factors behind RD in southwest China. Tang *et al*.^25^ identified soil calcium to be a possible driving element of RD progress in southwest China. However, even for those review works literally aiming at southwest China^2,24^, the macrosystem ecological^26^ (abbreviated as macroecological, hereafter) pattern of RD or its deciding mechanisms have not been explored in a holistic way yet. The reasons are that the traditional approaches based on field surveying are inefficient to cover a so large area^2^, and the tremendous complexity of karst geomorphology in this region makes the popular remote sensing-based solutions that tend to be available for large areas limited by large uncertainties^27^. Overall, for southwest China, the knowledge about its macro-scale ecological patterns of RD distribution and development has been in shortage, and this ill status now restricts making proper macroscopic polices for its environmental protection.

**Figure 2:**
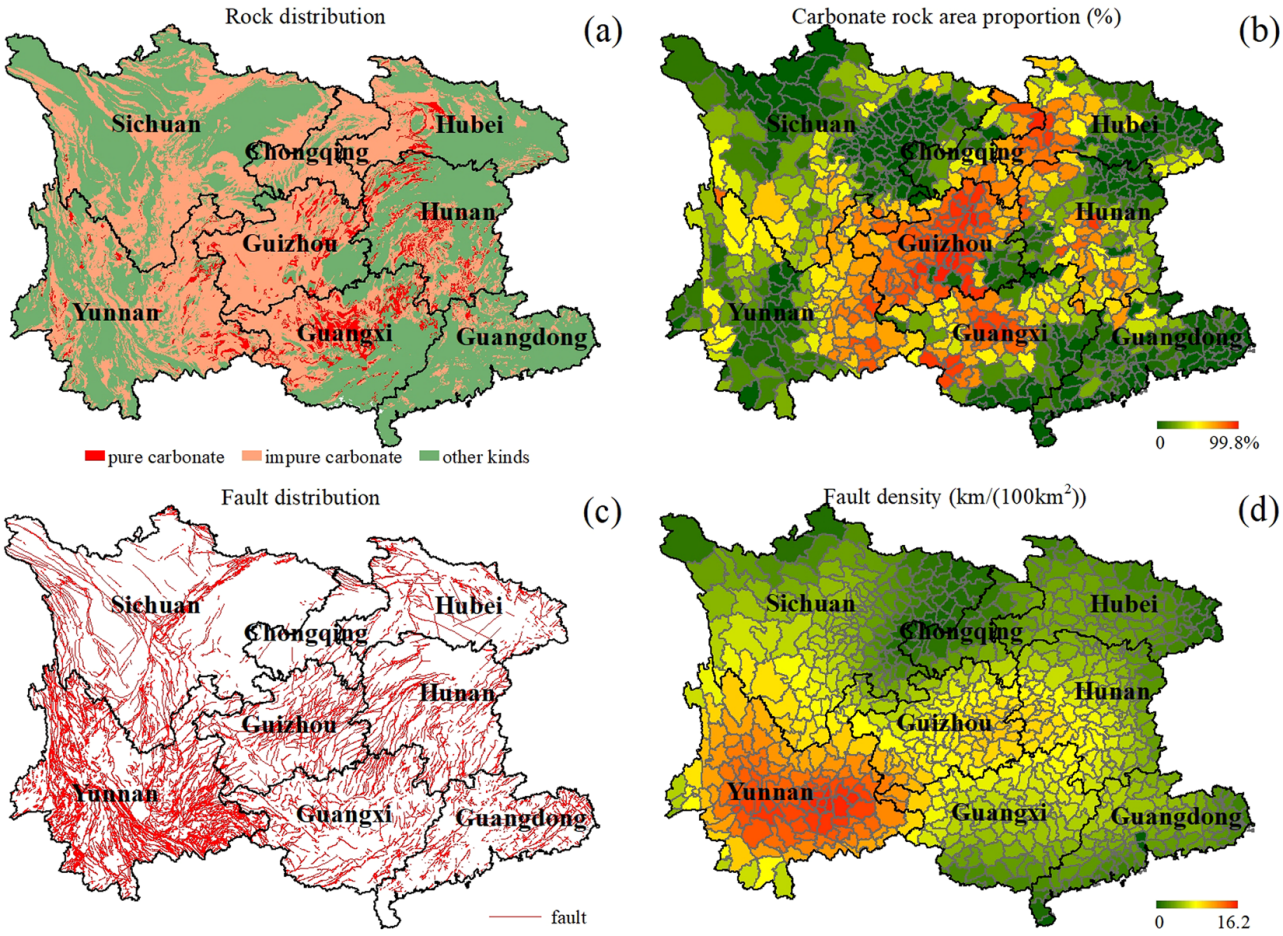
The colored maps of the considered two geologic factors in southwest China: (**a**) the spatial distribution of the three classifications of bedrocks, (**b**) the spatial pattern of carbonate rock distribution, in terms of carbonate area proportion per county, (**c**) the spatial distribution of faults, and (**d**) the spatial pattern of fault distribution, in terms of fault density per county.

**Figure 3:**
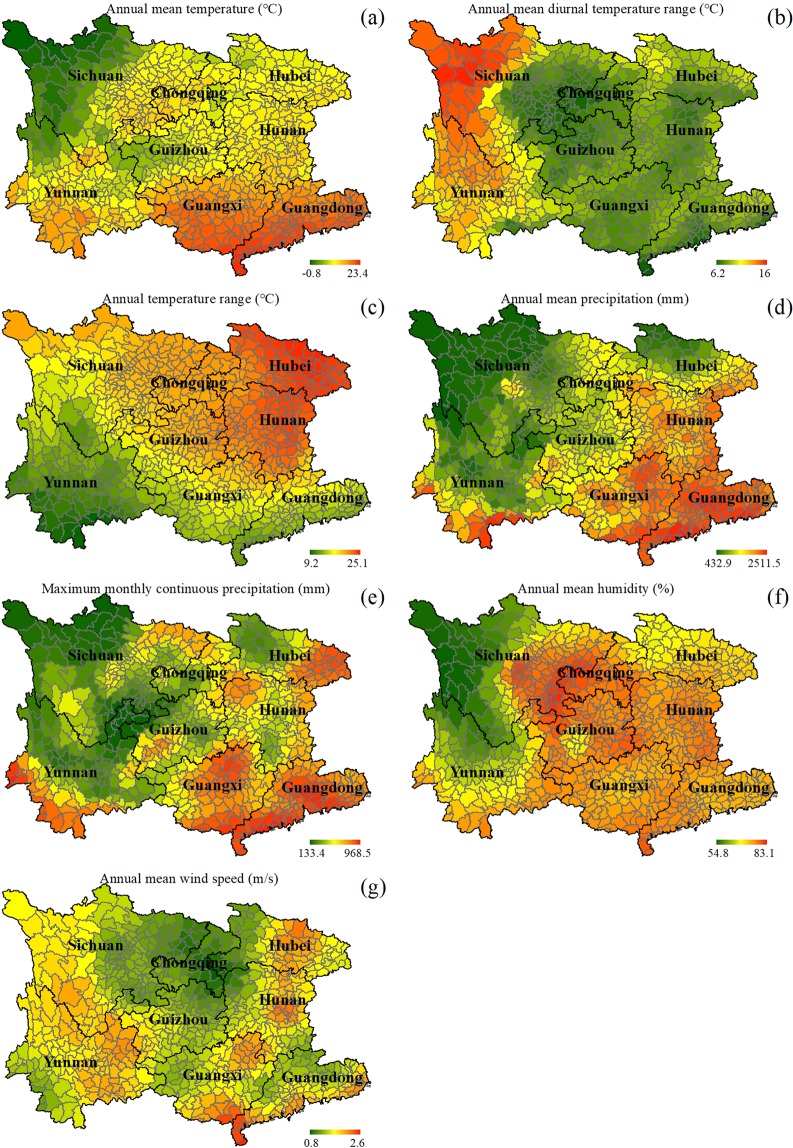
The colored maps of the regarded climatic factors in southwest China: (**a**) annual mean temperature (°C), (**b**) annual mean diurnal temperature range (°C), (**c**) annual temperature range (°C), (**d**) annual mean precipitation (mm), (**e**) maximum monthly continuous precipitation (mm), (**f**) annual mean humidity (%), and (**g**) annual mean wind speed (m/s).

**Figure 4:**
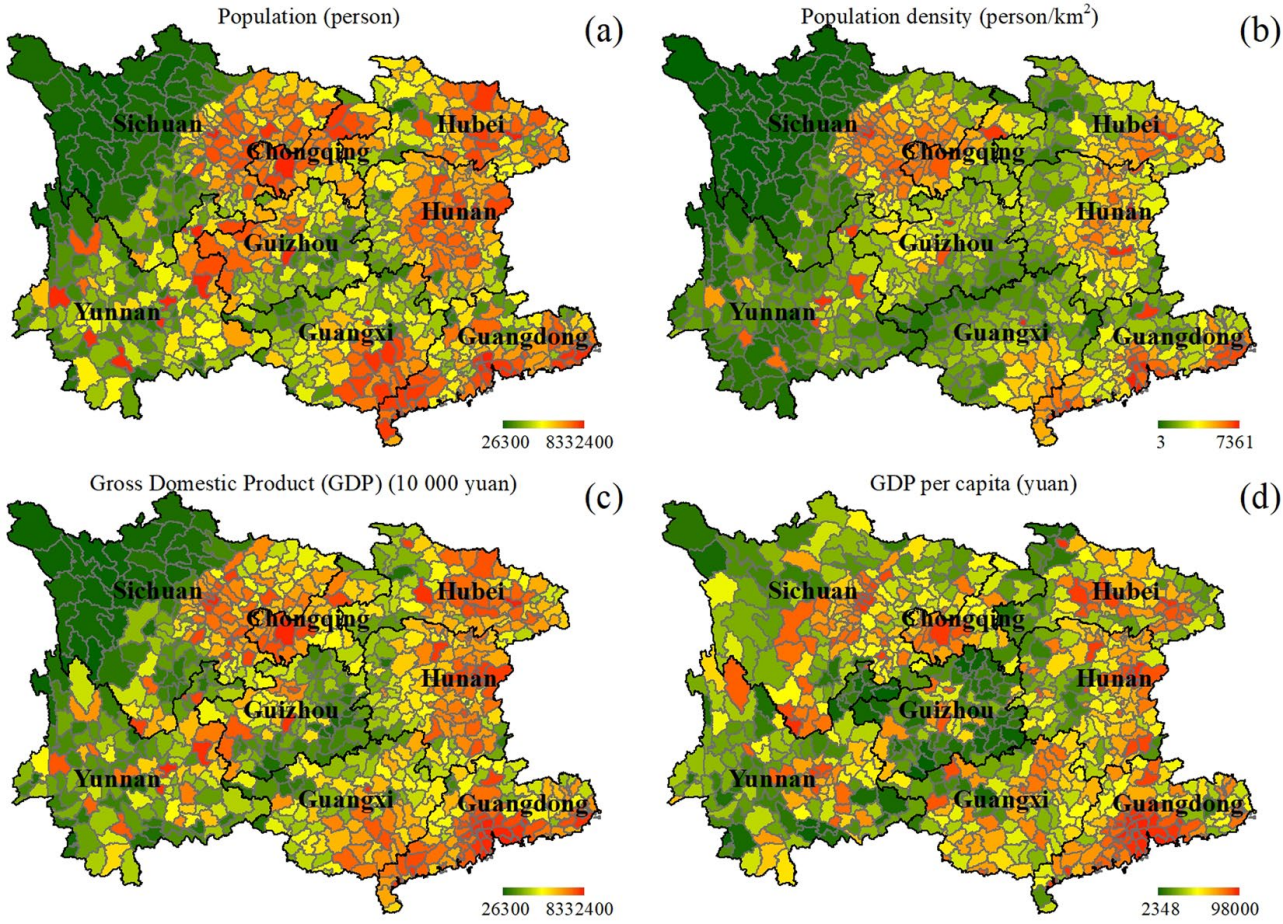
The colored maps of the concerned four anthropogenic factors in southwest China: (**a**) population (person), (**b**) population density (persons/km^2^), (**c**) gross domestic product (GDP) (10 000 yuan), and (**d**) GDP per capita (yuan/person) in each county.

To address this gap, this study alternatively took county as the unit to statistically probe the macroecological relationships between the 2008-censused distributions of county-level RD in southwest China^28^ and its potential impact factors. Southwest China is one of the three largest continuous karst regions in the world^28^, and it also shows the highest diversity of karst landscapes and the highest complexity of environment conditions^28^. Consequently, it is appropriate to choose southwest China as the study area for analyzing the complicated macroecological characteristics of RD. With the data of its RD distribution, also including the datasets of the slight-, moderate-, and severe-level scenarios (Fig. 1a–d, respectively), and the data of three typical categories of potential impact factors (geologic, climatic, and anthropogenic) (Figs. 2–4) piled up and then analyzed, this study aimed to (1) figure out the characteristics of the spatial distributions for both the RD scenarios at different severity levels and the impact factors, and (2) reveal the leading type of impact factors controlling the macroscopic pattern of RD distribution at each severity level and their contributions. The findings will be in favor of adding some basic knowledge as the references for further solving the mystery of how various impact factors influencing the macroecological patterns of RD formation and evolution in southwest China in the next-step works.

## Results

### Macroscopic characteristics of RD in spatial pattern

**Figure 5:**
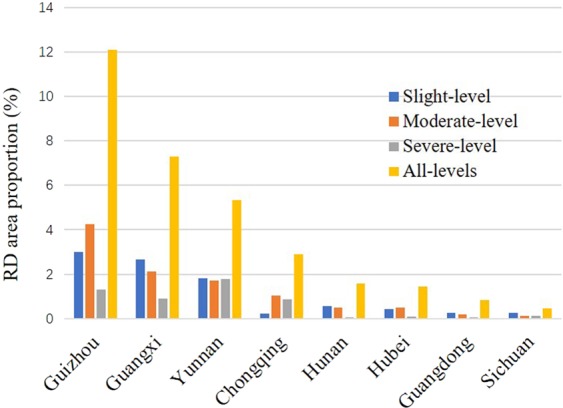
Boxplots of RD area proportion per county for the eight province-level municipalities in southwest China, derived by statistics in terms of slight-, moderate-, and severe-level, as well as their totality (all-levels).

Spatial statistics suggested that RD was distributed in every province-level municipality in southwest China, with the specific area proportions derived as 12.09% in Guizhou, 7.30% in Guangxi, 5.32% in Yunnan, 2.90% in Chongqing, 1.59% in Hunan, 1.44% in Hubei, 0.85% in Guangdong, and 0.45% in Sichuan in a descending order (Fig. 5). But in terms of the sum of RD coverage, the three leading provinces are Guizhou, Yunnan, and Guangxi, particularly in their southwest, east, and northwest, respectively (Fig. 1a). Spatial statistics also indicated that at the county level, RD is distributed in 66.06% of the counties in southwest China, with the largest RD area proportion per county reaching 44.10%. For the whole southwest China, the average RD area proportion per county is 3.60%. Overall, in view of macroscopic pattern, the spatial distribution of RD occurrences in southwest China is like a bird just spreading its two swings, taking over the central north-south axis of the whole region but heading toward the northeast direction to some extent.

Specifically for the RD distributions at the prescribed severity levels, their macroscopic patterns in southwest China are similar but shrink in area from the slight to severe level. First, the occurrences of the slight-level RD are primarily clustered in the central and south parts of Guizhou and the central part of Guangxi. Totally, the slight-level RD occurs to 64.36% of the counties in southwest China, with the largest RD area proportion per county reaching 25.32%, and its mean RD area proportion per county is 1.78%. Next, the moderate-level RD is mainly distributed in the south central part of Guizhou, the east part of Yunnan, and the central and west parts of Guangxi. The RD landscape of this level is distributed in 62.27% of the counties in southwest China, with the largest RD area proportion per county decreasing to 21.79%, and its average RD area proportion per county reducing to 1.15%. Finally, the severe-level RD is merely scattered in the east part of Yunnan. The RD lands at this level appear in 53.52% of the counties across southwest China, with the largest RD area proportion further decreasing to 17.70%, and its average RD area proportion cuts down even to 0.66%.

### Macroscopic characteristics of impact factors in spatial pattern

#### Geologic factors

The first geologic factor of carbonate rock in southwest China is primarily distributed in the intersection of Yunnan, Guizhou, and Guangxi, also stretching to the joint region between east Chongqing and west Hubei (Fig. 2a). The macroscopic spatial pattern of carbonate rocks in terms of area proportion in each county (Fig. 2b) is quite like the scenario of RD (Fig. 1a). Correlation analyses showed that carbonate rock and RD in area proportion per county has an obvious statistically significant positive correlation (R = 0.68, p < 0.0001), and this inference is also valid for the RD scenarios at the different severity levels (slight-level: R = 0.69, p < 0.0001; moderate-level: R = 0.62, p < 0.0001; severe-level: R = 0.42, p < 0.0001).

The second geologic factor of fault occurs widely over southwest China, with its densest distributions locating in Yunnan (Fig. 2c). The macroscopic spatial pattern of faults in terms of fault density in each county is decreasing from southwest to northeast (Fig. 2d), intuitively with a large difference from the scenario of RD (Fig. 1). Correlation analyses told that there is a statistically significant positive correlation (R = 0.38, p < 0.0001) between fault density and RD area proportion per county. As regards to the RD scenarios at the different severity levels, this rule still comes true (slight-level: R = 0.39, p < 0.0001; moderate-level: R = 0.35, p < 0.0001; severe-level: R = 0.30, p < 0.0001).

#### Climatic factors

In southwest China, the first climatic factor of annual mean temperature in macroscopic spatial pattern is decreasing from southeast to northwest in a relatively smooth way (Fig. 3a). Correlation analyses suggested that no statistically significant correlation (R = −0.056, p = 0.12) exists between this climatic factor and RD area proportion per county, nor do the peculiar RD scenarios at the three severity levels.

Next, annual mean diurnal temperature range in spatial pattern is dropping from west to east, with a distinctive step change (Fig. 3b). The western part of the study region possesses a high diurnal temperature range, while the central and east parts demonstrate relatively smaller temperature difference between day and night. Correlation analyses indicated that there is no statistically significant correlation (R = −0.062, p = 0.088) between this climatic factor and RD area proportion per county, neither do the RD scenarios at the three severity levels. However, a weak negative correlation can still be distinguished in the RD scenario at the slight severity level (R = −0.12, p = 0.0009).

The third climatic factor of annual temperature range in spatial pattern is increasing from southwest to northeast in a smooth way (Fig. 3c). Correlation analyses showed that there is a statistically significant negative correlation (R = −0.14, p < 0.0001) between this climatic factor and RD area proportion per county, similar with the slight- and moderate-level RD scenarios (slight-level: R = −0.23, p < 0.0001; moderate-level: R = −0.13, p = 0.0003) but different from the severe-level RD situations (R = −0.062, p = 0.086).

Annual mean precipitation in macroscopic spatial pattern is decreasing from southeast to northwest, but with a local area as an exception to this rule in the southwest Yunnan (Fig. 3d). After correlation analyses, no statistically significant correlation (R = −0.054, p = 0.13) between this climatic factor and RD area proportion per county emerged, nor for the three kinds of RD sub-scenarios.

Maximum monthly continuous precipitation in macroscopic spatial pattern is decreasing from southeast to northwest, but with several local areas such as southwest Yunnan, northeast Sichuan, and south Hunan as exceptions (Fig. 3e). Correlation analyses revealed that there is no statistically significant correlation (R = −0.099, p = 0.006) between this climatic factor and RD area proportion per county, even worse for the slight- and moderate-level RD scenarios. However, for the severe-level RD situation, there exists a weak negative correlation (R = −0.14, p = 0.0002).

Annual mean humidity in macroscopic spatial pattern is dropping from east to west, with a distinctive step change along the imaginary line drawn from central Sichuan to east Yunnan (Fig. 3f). The high values of this climatic factor mainly appear in Chongqing and east Sichuan. Correlation analyses found no statistically significant correlation (R = 0.034, p = 0.35) between this climatic factor and RD area proportion per county, and nor did for the three RD scenarios at different severity levels.

Annual mean wind speed in spatial pattern is a little approximate to a ‘U’ shape. The low values of annual mean wind speed in southwest China appear in the right north of the whole study region, including Chongqing, east Sichuan, west Hubei, west Hunan, and north Guizhou. Correlation analyses concluded that there is no statistically significant correlation (R = 0.016, p = 0.66) between this climatic factor and RD area proportion per county, and the same effects occurred to the three RD sub-scenarios at the slight, moderate and severe levels.

#### Anthropogenic factors

The first anthropogenic factor of population number in macroscopic spatial pattern is in a clustered way, i.e., each of the province-level municipalities presenting one or more clusters (Fig. 4a). Correlation analyses inferred that no statistically significant correlation (R = −0.079, p = 0.029) exists between this anthropogenic factor and RD area proportion per county, nor do the three RD scenarios at different severity levels.

For the second anthropogenic factor of population density, its clustered spatial pattern is more obvious, namely, the clustering centers can be more easily recognized in each province (Fig. 4b). Correlation analyses told that there is a statistically significant negative correlation (R = −0.13, p = 0.0002) between this anthropogenic factor and RD area proportion per county, and this rule is also roughly valid for the three RD sub-cases (slight-level: R = −0.13, p = 0.0003; moderate-level: R = −0.12, p = 0.001; severe-level: R = −0.086, p = 0.018).

In southwest China, GDP in macroscopic spatial pattern briefly obeys the characteristics of population number in each county, also with the approximate clustering centers (Fig. 4c). Correlation analyses exposed that no statistically significant correlation (R = −0.080, p = 0.026) can be detected between this anthropogenic factor and RD area proportion per county, nor can for the three peculiar RD sub-scenarios.

GDP per capita in macroscopic spatial pattern is like a mirrored one against the pattern of RD (Fig. 4d). Correlation analyses presented that there is a statistically significant negative correlation (R = −0.19, p < 0.0001) between this anthropogenic factor and RD area proportion per county. The three RD sub-scenarios at the different severity levels work in the same way. The reason is that the larger the scope of RD distribution is, the more people’s livelihood is affected, and so, people tend to gather in areas that are easier to earn a living.

### Macroecological relationships between RD and impact factors

**Figure 6:**
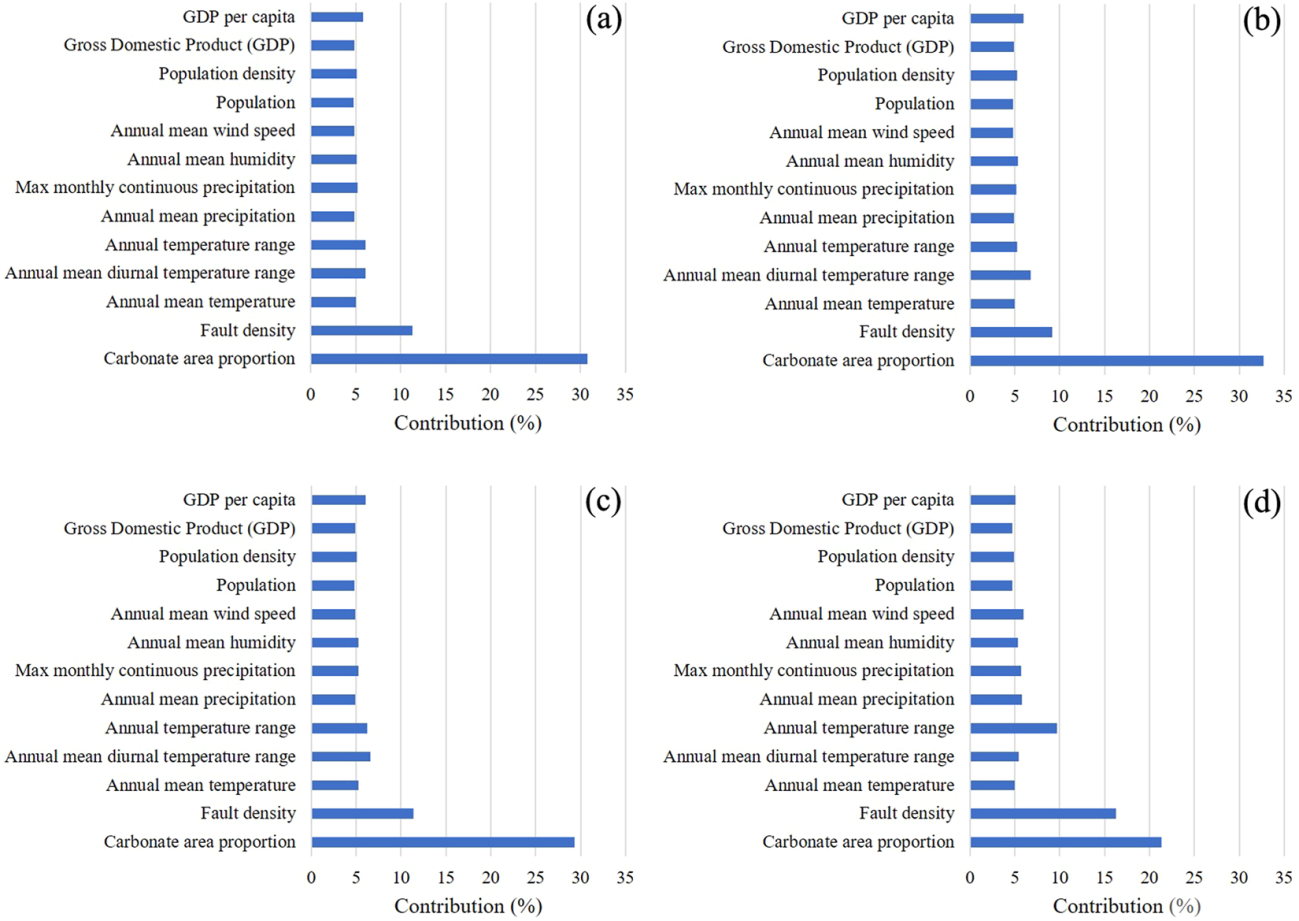
Contributions of the prescribed 13 impact factors to RD in southwest China, derived in terms of (**a**) all-levels RD and its (**b**) slight-, (**c**) moderate-, and (**d**) severe-level scenarios.

In exploring the macroecological relationships between the RD distribution in southwest China and the three kinds of impact factors, dominance analyses derived the contributions of the latter ones in influencing the former one (Fig. 6a). The predominant five impact factors in a descending order include carbonate rock area (contribution = 30.77%), fault density (11.29%), annual mean diurnal temperature range (6.14%), annual temperature range (6.10%), and GDP per capita (5.84%). This suggests that at the macro scale, the most critical kind of influencing forces to RD is geological factors.

For the slight-, moderate- and severe-level RD sub-scenarios, the key five impact factors were individually determined based on the same approach of dominance analysis and listed in a descending order: (1) slight-level RD: carbonate rock area (32.66%), fault density (9.17%), annual mean diurnal temperature range (6.79%), GDP per capita (6.01%), and annual mean humidity (5.35%) (Fig. 6b); (2) moderate-level RD: carbonate rock area (29.33%), fault density (11.4%), annual mean diurnal temperature range (6.57%), annual temperature range (6.27%), and GDP per capita (6.06%) (Fig. 6c); (3) severe-level RD: carbonate rock area (21.33%), fault density (16.27%), annual temperature range (9.70%), annual mean wind speed (5.98%), and annual mean precipitation (5.82%) (Fig. 6d).

The results indicated that for the four concerned scenarios different arrays of the factors take the predominant roles in drawing the macroecological patterns of RD in southwest China. However, there are still some common rules that can be derived. By and large, the carbonate rock area- and fault density-represented geologic factors play the first role; the climatic and anthropogenic factors take the second and third places; the anthropogenic factors show their effects in a weakening way from the slight-level RD scenario to the severe-level one, and this mode is evidenced by the factor sequence obtained in the all-levels RD scenario.

Overall, we concluded that geologic factors leadingly drew the macroecological patterns of RD in southwest China, for the four scenarios of slight-, moderate-, and severe-level RD as well as their totality. This inference seems to be contrasting with the popular awareness that anthropogenic factors such as human activities and land uses shall be first responsible for the RD-relevant losses^1,2,6,11,19^. The discussions of this differing-from-tradition inference, regarding its origin, physics, uncertainties, future works, and implications, were carried out as follows.

## Discussions

The discrepancy between the inferences from a lot of earlier studies^2^ and those from this study critically originates from their difference in point of interest. Earlier studies focused on the driving power of various impact factors in changing RD statuses^19^, while this study highlighted the macroecological relationships in various impact factors leading to RD statuses. In consequence, in the former scenario, the RD-related variables are often characterized by their variations during limited periods, and that is why anthropogenic effects that have kept strengthening along with industrial development have been often detected as the main driving forces^29^; but for the latter scenario, RD is mostly characterized by its statuses, and hence, macroecological analyses showed more about the long-run instead of short-term and deciding instead of changing effects of various impact factors on RD. This theoretical explanation can somehow reduce the confusions possibly caused by this inference controversy.

For further understanding of this inference difference, the effects of the impact factors on the macroscopic spatial pattern of RD in southwest China and their physical foundations are individually elucidated as follows. As the basis of topographical evolution, geologic factors, here, proved to play the predominant role in drawing the macroecological pattern of RD. The reason is that carbonate rocks often relate to a fragile and vulnerable environment, which is susceptible to deforestation and, further, RD^2^. As found by earlier studies, the estimated soil formation rate on average is ~11 tons per square kilometer annually in the karst areas of southwest China^30^, while purple soil corresponds to a formation rate of 800–1200 tons per square kilometer per year in the non-carbonate rock area of the Sichuan Basin^31^. When soil loss is severe enough to reach a certain threshold, RD may become irreversible^32^. These processes determined the regional pattern of RD, as evidenced by the approximate inference based on the data from land resource surveys^33^. Other natural processes involving climatic and hydrologic factors can exacerbate the occurrence of RD. For example, the shallow karst zones cannot maintain their water levels, due to the high permeability of karst feature. Thus, the storage capacity of water in the soil layers is low, which results in the deficiency of soil moisture for vegetation growth. In addition, areas with less dense surface streams tend to suffer from more soil erosions and, subsequently, a higher tendency toward RD^34^. As regards to anthropogenic factors, the focused four variables mostly behaved with a negative correlation coefficient here. This is due to that in macroecological pattern, human beings still abide by the livable conditions of the landscape. From this aspect, anthropogenic effects are trivial in affecting the macroecological pattern of RD. However, as noted that disturbances from a large variety of human activities shall be ultimately responsible for RD wherever it has occurred^2^, the effects of various anthropogenic factors on RD still need to be highlighted and explored in the future works.

The uncertainties lying in the results of this study also need to be discussed. Although the proposed scheme of county-based RD analysis can innovatively solve the traditional issue of RD macroecological analysis, it, from the origin, can be confirmed as a critical source of introducing uncertainties into the results. In addition, the three kinds of impact factors present different effect phases, different effect spatial-scales, and different effect modes. For the study area, geologic factors have effectively played their roles for millions of years, climatic factors have made their effects for thousands of years, while anthropogenic factors have only showed their power for hundreds of years^2^. This difference, no doubt, may render the inferences in RD macroecology with large uncertainties. Moreover, many other kinds of disturbance factors such as land use^20^, slope^35^, elevation^36^, landform type^19^, or hydrology (e.g., river density)^29^ have not been examined in this study. As earlier works found, the huge relief in topography and steep land surface slopes created by multiple tectonic movements can provide kinetic energy for overland flow, which enhances soil erosion and karstification further^37^. Such effects on RD may cause uncertainties as well. Thereby, such factors and their effects possibly causing the uncertainties in the macroecological studies of RD need to be paid more attention in the following works.

The future work in this direction is to study more comprehensive mechanisms under this newly-highlighted macroecological framework of RD pattern. First, the scaling gap between the macroecological pattern and fine-scale ecological pattern^2^ of RD needs to be bridged. This bridging process may involve varying causes and uncertainties at different scales, and this means more and more detailed analyses will be extended from this study in the future. The next-step works need to probe all of the aspects of RD macroecology. For example, what kinds of macroecological effects will be derived from the phenomena that soil temperatures in southwest China have showed increasing trends over the last couple of decades^38^ and the frequency of extreme droughts has intensively increased in the past 50 years in many parts of southwest China^39^. Such explores will surely expose more scientific information about RD mechanisms and evolutions.

Ultimately, the findings in this study can help to guide making more appropriate policies for coping with the RD-typed land degradations. As we know, the implementation of various policies on environmental restorations mainly lies on regulating people’s activities. However, the negative correlation of anthropogenic factors with RD suggested that people in residence still obey the beneficial macroscopic mode, as a mirrored one for this inverse geomorphology. In other words, such policies in effect possess the inherent limitations to the human-reaching ranges, and consequently, the traditional policies, substantially, cannot effectively handle the RD issues in the widely-spread areas with few populations in southwest China. To solve such problems, administration shall consider making more macroscopic policies with the measures adaptable to carbonate rock land covers, by following the macroecological patterns of RD and its impact factors found in this study. For example, regional planning of land uses shall leave the karst landscapes with more spaces for natural preservation, and aerial seeding of natural plants growing better in the karst landforms shall be more arranged in times for those human far-reaching areas. Such plans shall be adaptable for the other RD regions in the world.

## Materials and Methods

### Study area

Southwest China (Fig. 1) (1.91 million km^2^, approximate to Indonesia – the 14^th^ country in area) has about 0.54 million km^2^ of exposed/outcropped carbonate rock areas. The RD area proportion reaches about 28% of the whole study area, and hence, studying the characteristics of RD in this massive region is a matter of cardinal significance. Further, the significance is intensified when regarding the high diversity of RD statuses existing in southwest China. This high diversity can be featured by RD severity, often classified as three levels in China^2^: (1) severe-level RD: exposed rocks compose the coverage larger than 70%; (2) moderate-level RD: exposed rocks between 50% and 70%; (3) slight-level RD: exposed rocks between 30% and 50% (Note that exposed rocks less than 30% as no RD). This rule was also abided by this study in exploring the links between RD and impact factors. More detailed information about RD in southwest China can refer to^2^.

### Data preparation

First, we used the 2008-censused county-level RD data in southwest China^28^ for this study. In each county, the RD-relevant data were surveyed and sorted out in terms of slight-, moderate-, and severe-level RD area, and county area^28^. Based on this dataset, the variable of RD area proportion per county and those at the separate RD severity levels were derived by dividing the related RD area by the county area and saved as the related maps (Fig. 1).

Two geologic factors of carbonate rock and fault were considered in this study, and their data were derived from the 1:500 000 geologic maps of China (www.geodata.cn, accessed at 10 February, 2019). For carbonate rock, its situations were divided into three kinds: dolomite, limestone, dolomite interbedded with limestone, dolomite with limestone, and limestone with dolomite were divided into pure carbonate rock, carbonate rock with clastic rock, carbonate interbedded with clastic rock, and clastic rock with carbonate rock were divided into impure carbonate rock, and the other lithological forms were classified as other kind. Quantitatively, the definition of pure carbonate rock is the rock with carbonate content more than 90% while clastic rock content less than 10%, and impure carbonate rock refers to the rock with more than 10% carbonate rock content and more than 10% clastic rock content^28^. In accordance to this rule, the map of the three classifications of carbonate rocks (Fig. 2a) was generated, and the map of carbonate rock situations in terms of carbonate rock area proportion per county (Fig. 2b) was also derived in the same way as did for RD. For faults, their spatial distributions in southwest China were shown (Fig. 2c). The map reflecting fault distributions was derived (Fig. 2d) by the same means, but in terms of fault density per county. We defined this variable as the summed length of faults compared to the administrative area of the related county (km/(100km^2^)).

The climatic factors investigated in this study included annual mean temperature (°C), annual mean diurnal temperature range (°C), annual temperature range (°C), annual mean precipitation (mm), maximum monthly continuous precipitation (mm), annual mean humidity (%), and annual mean wind speed (m/s). Their 2008-related basic data were downloaded from China Meteorological Data Network (data.cma.cn, accessed at 10 February, 2019) and derived by statistics per county according to their corresponding literal definitions (Fig. 3). The anthropogenic factors regarded in this study comprised population (person), population density (persons/km^2^), GDP (10 000 yuan, i.e., the basic unit of RMB), and GDP per capita (yuan/person). Their 2008-related basic datasets were downloaded from the Yearbooks of the eight province-level municipalities (www.geodata.cn, accessed at 10 February, 2019), and their maps were derived (Fig. 4). Among, population density was quantified by dividing the population number with the related administrative area in each county, and GDP per capita was obtained by dividing the GDP with the related population in each county.

### Spatial pattern analysis

#### Spatial statistics

The macroscopic spatial pattern of RD in southwest China was first analyzed by spatial statistics. Specifically, with county taken as the basic unit, the average RD area proportion in southwest China was derived by calculating the division between the sum of RD areas in each county and the sum of the administrative areas for all of the counties, and so did at the three severity levels. The same routine was operated for each of the province-level municipalities, also with their orders sorted out, and so did at each of the three severity levels. Then, the ratio of counties with RD occurring in southwest China was calculated to reflect its universality in a whole sense, and so did not only at each of the three severity levels but also in each of the province-level municipalities. Next, the largest RD area proportion per county was sought to mirror the severity of RD development risk across southwest China. Based on the software of ArcGIS (https://www.arcgis.com/, accessed at 10 February, 2019), all of these metrics were derived in order to reflect the primary characteristics of RD in the study area.

### Artificial interpretation

The macroscopic spatial pattern of RD in the study area was then examined by artificial interpretation. This approach is often implemented by setting the variable with varying colors or grayness correspondingly in mapping its related spatial distribution^40^, and then, people with the relevant knowledge can visually interpret the generated map and artificially infer the characteristics of its spatial pattern from the spatial variation of colors or grayness. Here, for RD and the 13 impact factors, both color setting and artificial interpretation were operated based on ArcGIS (https://www.arcgis.com/, accessed at 10 February, 2019), in order to derive the macroscopic characteristics of their spatial patterns, e.g., clustering or scattering, smooth change or step change, and bird shape or snake shape, in southwest China.

### Attribution analysis

#### Correlation analysis

To quantitatively show the potential link between RD and each of the prescribed impact factors, Pearson’s correlation analysis^41^ was first operated to evaluate the strength of the relation between the concerned two variables. A high correlation (statistically characterized by high correlation coefficient R and low p-value) means that the two variables have a strong relationship, while a weak correlation (low R and high p-value) means that the variables are hardly related^42,43^. In this study, by taking the county as the basic unit, we examined the relationships between RD and each of the 13 impact factors by inputting them as the variables into the Correlation Analysis module of the software Matlab (www.matlab.cn, accessed at 10 Feburary, 2019), and the analyses were operated for the four targeted scenarios. The results of such exploratory analyses can simply reflect the power of the impact factors in influencing the macro-spatial pattern of RD in southwest China.

#### Dominance analysis

The approach of dominance analysis proposed by Budescu^44^ was used to analyze the macroecological contributions of the prescribed impact factors to the spatial pattern of RD in southwest China. Dominance analysis is a statistical method used to determine the order of “dominance” or “relative importance” of predictors in an established linear regression model and for a given set of p predictor variables^45,46^. Here, the specific scheme was set up as follows. First, by county, RD at each severity level or their totality was set as the dependent variable, and all of the 13 impact factors were set as the predictor variables. Then, the two sets of variables were input to the Dominance Analysis module in the DPS software^47^, and the analyses were implemented for the four aimed scenarios. Finally, the coefficients of the established linear regression model marked the relative importance, namely, the contributions, of the impact factors. Based on their comparisons, people can derive which factors primarily decide the macroecological pattern of RD in the study area.
